# Effect of Visualized Respiratory Training on Patient Cooperation and Image Quality in Gadoxetic Acid Disodium‐Enhanced Liver MRI: A Randomized Controlled Trial

**DOI:** 10.1002/jmri.70423

**Published:** 2026-06-23

**Authors:** Li Mei, Xin Luo, Bingyan Wang, Junfeng Wang, Chao Huang, Xuemei Hu, Qin Li, Wenyan Zhang

**Affiliations:** ^1^ Department of Radiology, Tongji Hospital, Tongji Medical College Huazhong University of Science and Technology Wuhan China; ^2^ Department of Nursing, Tongji Hospital, Tongji Medical College Huazhong University of Science and Technology Wuhan China

**Keywords:** dynamic contrast‐enhanced liver MRI, gadoxetic acid disodium (Gd‐EOB‐DTPA), image quality, patient cooperation, visualized respiratory training

## Abstract

**Background:**

Respiratory motion artifacts impair diagnostic accuracy in gadoxetic acid disodium (Gd‐EOB‐DTPA)‐enhanced liver MRI. Conventional verbal pre‐scan breathing training often yields inconsistent patient cooperation and image quality, creating an urgent clinical need for optimized training methods.

**Purpose:**

To evaluate the impact of a visualized respiratory training method integrated into clinical workflow on patient cooperation, image quality, and examination efficiency for Gd‐EOB‐DTPA‐enhanced liver MRI.

**Study Type:**

Prospective, single‐center, randomized controlled trial.

**Population:**

One hundred and eighty‐four patients (135 males; mean age 53.5 ± 13.3 years) scheduled for first‐time Gd‐EOB‐DTPA‐enhanced liver MRI.

**Field Strength/Sequence:**

3 T; Dynamic contrast‐enhanced imaging was performed with a 3D fast spoiled gradient‐echo sequence, covering pre‐contrast, arterial, portal venous, transitional, and hepatobiliary phases.

**Assessment:**

Primary outcome was patient cooperation rate assessed by blinded MRI technologists. Secondary outcomes included motion artifact scores (rated by three blinded radiologists), Signal‐to‐Noise Ratio (SNR), Contrast‐to‐Noise Ratio (CNR), transient severe motion (TSM) incidence, and examination duration.

**Statistical Tests:**

Student's *t*‐test, Mann–Whitney *U* test, Chi‐square test, and Gamma correlation; two‐tailed *p* < 0.05 indicated significance.

**Results:**

The experimental group (85 patients) had a significantly higher full cooperation rate (56.5% [48/85] vs. 27.3% [27/99], OR = 3.46, 95% CI: 1.87, 6.40, *γ* = 0.406), significantly lower motion artifact scores in arterial/portal venous/transitional phases, and significantly higher arterial and transitional phase SNR/CNR. The experimental group also showed significantly shorter preparation time (14.40 ± 2.36 vs. 16.16 ± 2.20 min; mean difference: −1.76 min; 95% CI: −2.42, −1.101). Scanning time did not differ significantly between groups (24.60 ± 1.60 vs. 24.89 ± 2.21 min; *p* = 0.329). TSM incidence did not differ significantly between groups (1.18% vs. 5.05%, *p* = 0.219).

**Data Conclusion:**

Visualized respiratory training significantly improves patient cooperation, image quality, and workflow efficiency in Gd‐EOB‐DTPA‐enhanced liver MRI, with no additional resource requirements supporting its routine clinical adoption.

**Evidence Level:**

2.

**Technical Efficacy:**

Stage 1.

## Plain Language Summary

Liver MRI scans often become blurry when patients cannot hold their breath correctly, which can reduce diagnostic accuracy, while standard verbal breathing training has inconsistent results. We randomly assigned 184 patients to either standard verbal training or a new visualized training system that shows patients their breathing in real time. We found this new training improved patient cooperation, produced clearer images, and shortened scan preparation time. This system can be easily added to routine clinical MRI practice without extra staff, space, or high costs, offering a simple way to improve liver MRI quality and efficiency.

## 1 Introduction

Dynamic contrast‐enhanced magnetic resonance imaging (DCE‐MRI) with gadoxetic acid disodium (Gd‐EOB‐DTPA) is routinely used for the diagnosis and characterization of focal liver lesions, particularly hepatocellular carcinoma (HCC), owing to its unique dual‐phase (vascular and hepatobiliary) enhancement properties [1, 2, 3]. However, the diagnostic utility of the critical arterial phase is frequently compromised by respiratory motion artifacts [4, 5, 6]. These artifacts, manifesting as blurring and reduced lesion conspicuous, can degrade both subjective image quality and objective metrics such as signal‐to‐noise ratio (SNR) and contrast‐to‐noise ratio (CNR), potentially leading to missed diagnoses or misinterpretations [7]. A particularly challenging form of this artifact is transient severe motion (TSM), which disproportionately affects the arterial phase [4, 5, 6]. Reported incidence rates for TSM in Gd‐EOB‐DTPA‐enhanced examinations range from 4.8% to 26.7% [6].

Pre‐scan respiratory coaching is an intervention sometimes employed to mitigate these artifacts. Effective breath‐holding can improve temporal resolution and spatial registration during dynamic acquisition [8]. Nevertheless, conventional training paradigms predominantly rely on unidirectional verbal instruction and subjective assessment by technologists (e.g., visual inspection or palpation) [9, 10]. This approach suffers from inherent limitations: a disconnect between the training environment and the actual scanner experience, a lack of objective, real‐time feedback for the patient, and inter‐operator variability in instruction and assessment quality. Consequently, patient comprehension, cooperation, and breath‐hold consistency are often suboptimal, resulting in variable and sometimes unsatisfactory training outcomes [9, 10].

Visual biofeedback systems present a promising avenue to overcome these limitations. By translating respiratory effort into intuitive, real‐time visual cues (e.g., waveforms), such systems can provide patients with immediate feedback on their breathing pattern, facilitating self‐correction and mastery of breathing commands. Prior research in related contexts suggests that visual aids can enhance procedural understanding, reduce anxiety, and improve adherence to medical instructions [11, 12, 13, 14, 15]. In imaging, preliminary studies indicate that tailored breathing instructions or simple feedback can reduce motion artifacts [16, 17]. However, a comprehensive, randomized evaluation of a structured visualized respiratory training program within the complete clinical workflow of Gd‐EOB‐DTPA‐enhanced liver MRI is lacking. Specifically, the integrated impact of such a system on patient cooperation during the actual scan, image quality (subjective and objective), and overall examination workflow efficiency remains to be conclusively determined.

Thus the purpose of this prospective randomized controlled trial was to evaluate the value of a novel visualized respiratory training method in patients undergoing Gd‐EOB‐DTPA‐enhanced liver MRI.

## 2 Materials and Methods

### 2.1 Participants

This prospective randomized controlled trial was conducted in the radiology department of a single tertiary hospital. Between June 2023 and October 2024, 184 consecutive patients scheduled for gadoxetic acid (Gd‐EOB‐DTPA)‐enhanced dynamic liver MRI were prospectively enrolled. The study protocol was approved by the Institutional Review Board of Tongji Hospital, Tongji Medical College, Huazhong University of Science and Technology (Ethics Approval No. TJ‐IRB20230705), and written informed consent was obtained from all participants.

Participants were eligible if they (a) were aged 18 years or older; (b) were scheduled for a first‐time Gd‐EOB‐DTPA‐enhanced liver MRI; (c) had no contraindications to contrast MRI (e.g., a non‐MRI‐compatible cardiac pacemaker, or a known allergy to gadolinium‐based contrast agents); and (d) provided written informed consent. Enrolled participants were considered discontinued from the study if any of the following occurred: (a) inability to comprehend or follow instructions during the training; (b) development of an acute clinical condition that precluded safe continuation; (c) emergence of severe claustrophobia or other psychological distress preventing completion of the MRI; or (d) voluntary withdrawal of consent at the patient's request. Figure 1 presents the detailed participant screening and enrollment process of the present study.

**FIGURE 1:**
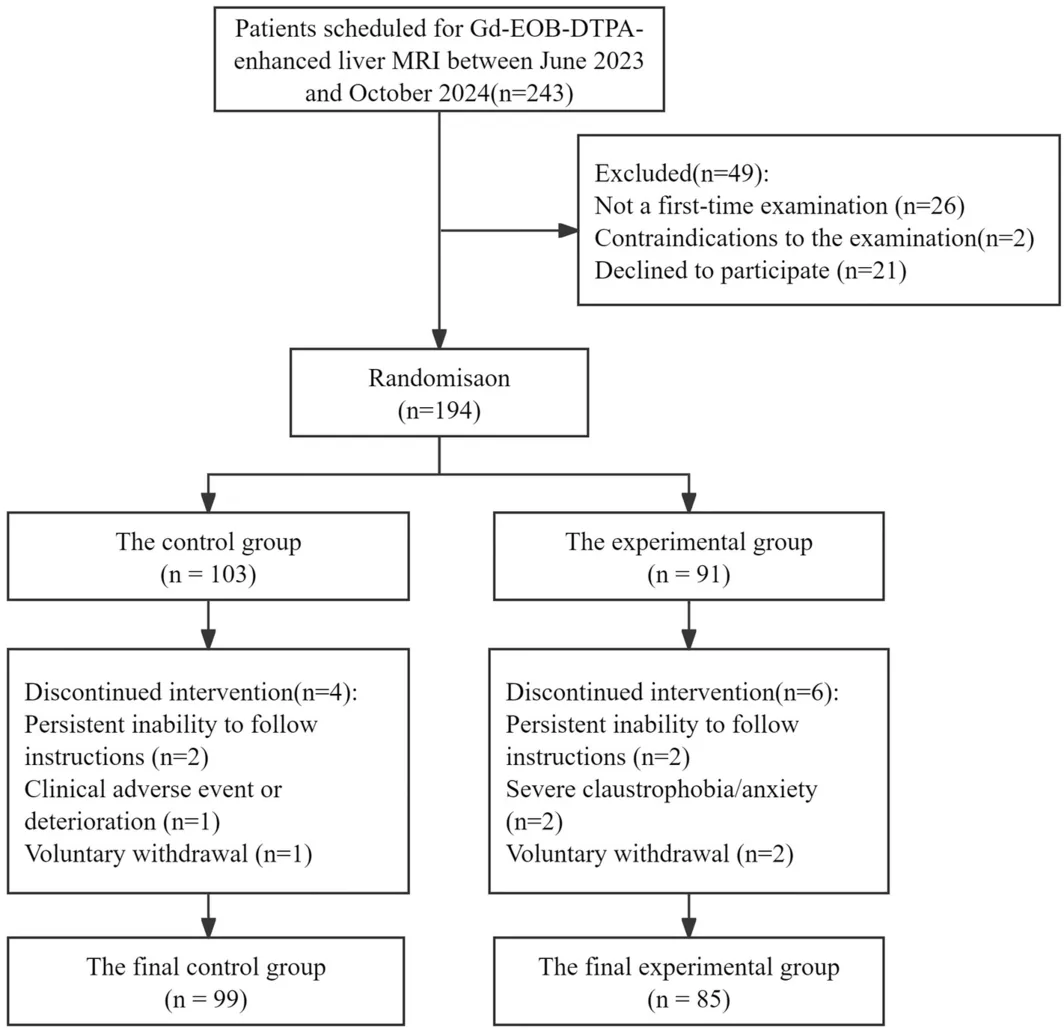
Flowchart of patient enrollment, exclusion, and group allocation.

### 2.2 RCT Allocation

Randomization was implemented using a simple method based on the parity (odd or even) of the last digit of each patient's pre‐assigned imaging examination number. Imaging examination numbers were assigned sequentially by the hospital's radiology information system at the time of scheduling, independently of and prior to study enrollment. Blinding was rigorously implemented in this study. Patients and MRI technologists responsible for evaluating scan cooperation were blinded to group assignment. All participants underwent Gd‐EOB‐DTPA‐enhanced liver MRI for the first time with no prior experience of respiratory training, and training was performed by dedicated research nurses in a separate preparation room, which prevented patients from distinguishing between training protocols. In addition, radiologists performing qualitative image assessment were fully blinded to group allocation. All images were anonymized with all identifying information removed; sequence labels were standardized without group‐specific information, and image datasets from both groups were randomized and intermixed before independent review, with no access to clinical or training‐related data.

### 2.3 Training Method

The visualized respiratory training system comprised a hardware assembly and a software interface (Figure 2). The core hardware included an adjustable abdominal belt and a mobile terminal. The belt, designed to fit various body sizes, contained embedded pressure sensors within its central deformable portion to detect respiratory‐induced abdominal wall movements and an integrated heart rate sensor. Signals from these sensors were transmitted to a microcontroller, converted to digital format, and then relayed wirelessly to the mobile terminal via Wi‐Fi or Bluetooth. The mobile terminal hosted a custom application that provided two primary functions: (a) real‐time biofeedback: It displayed intuitive, real‐time visual waveforms of the user's breathing pattern, offering immediate feedback; and (b) training protocol management: It featured an interactive interface for initiating different training modes (e.g., regular breathing, single breath‐hold, and serial breath‐holds).

**FIGURE 2:**
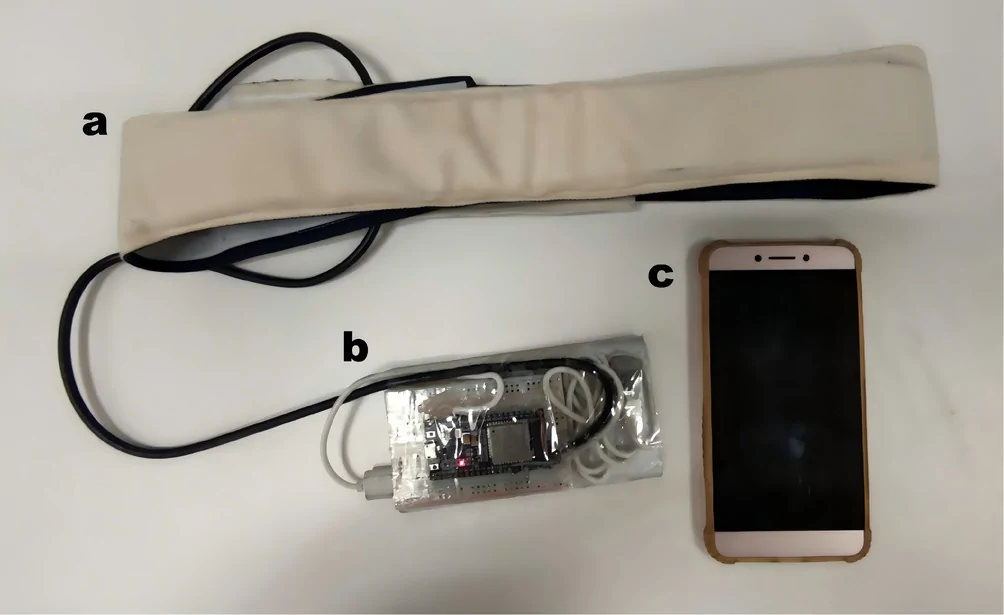
The visualized respiratory training device. (a) Abdominal belt. (b) Microcomputer. (c) Mobile terminal.

All participants received standardized pre‐training education in a private preparation room to mitigate anxiety and ensure comprehension (Figure 3). The session, conducted by a research staff member, covered MRI‐related information, examination precautions, breathing coordination techniques, potential sensations during contrast injection, and anticipated equipment noise levels. All training was performed with the patient in a seated position. The training protocol for both groups comprised three sequential stages: (1) regular breathing adjustment (1 min); (2) single breath‐hold (15 s); and (3) three consecutive breath‐holds. Training efficacy was assessed using either subjective (control group) or objective (experimental group) criteria, with participants proceeding to MRI only after meeting the respective standards. Participants who failed to meet any criterion received targeted corrective training for the specific deficient component until all standards were satisfied.

**FIGURE 3:**
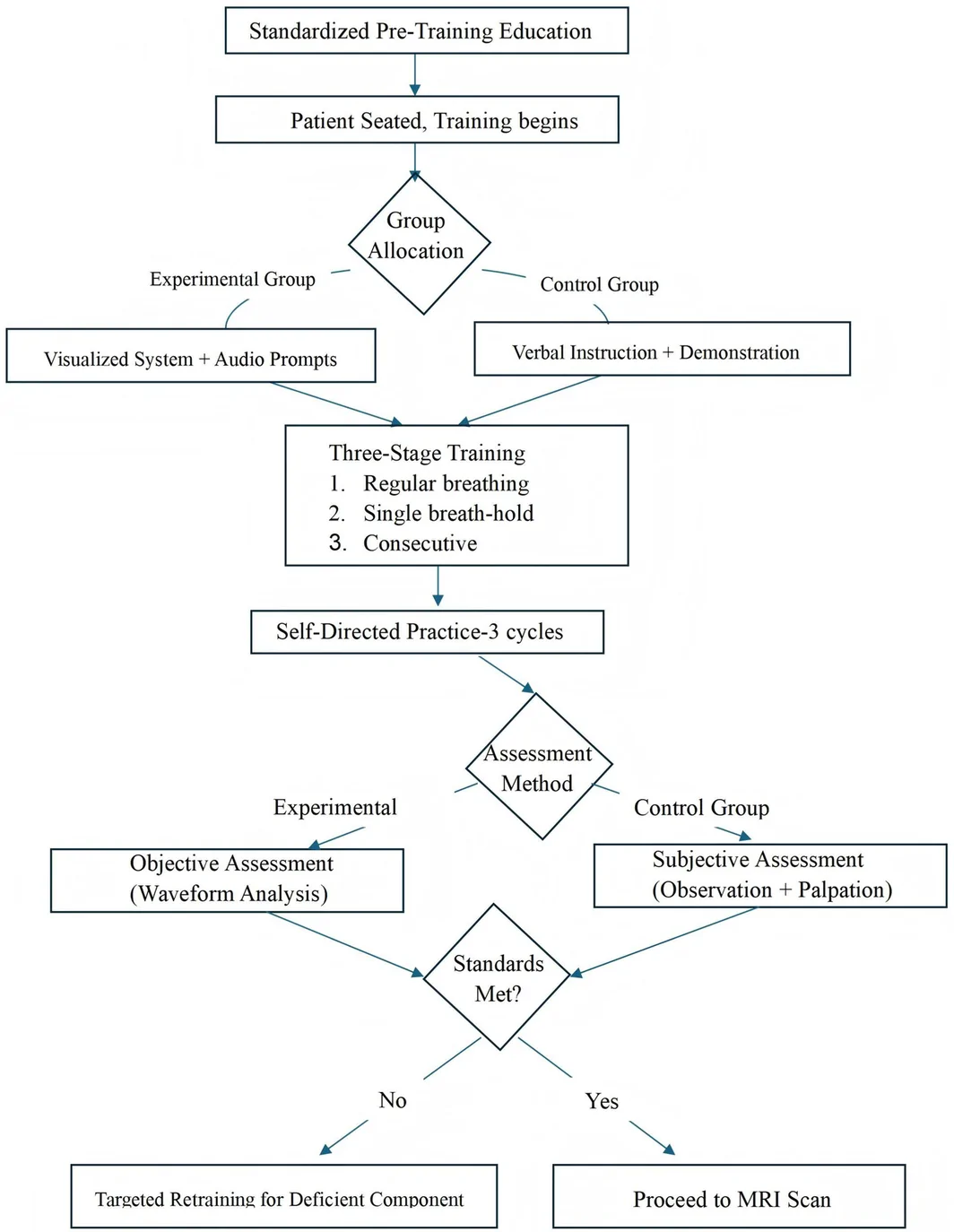
Flowchart of the respiratory training protocol.

#### 2.3.1 Control Group

Participants received conventional verbal instruction. The researcher guided the patient through one round of three‐stage breathing training and explained the key cooperation techniques and essentials. The patient then performed approximately three cycles of self‐directed practice (5 min). Training efficacy was assessed subjectively via observation and palpation based on: (a) regular breathing stability (steady rhythm, consistent abdominal excursion); (b) single breath‐hold compliance (absence of abdominal motion during 15 s); and (c) sequential breath‐hold capacity (successful completion of three consecutive breath‐holds).

#### 2.3.2 Experimental Group

Participants underwent training using the visualized respiratory training system. The researcher fitted the patient with an abdominal belt sensor and initiated the mobile terminal program. The patient was guided to follow the audio prompts from the respiratory training device to complete the three‐stage training protocol and was instructed to adjust their breathing in real time according to the respiratory waveforms displayed on the mobile terminal. After approximately three cycles of self‐guided practice (5 min), training efficacy was assessed objectively via waveform analysis based on: (a) stable regular breathing waveform (consistent frequency and amplitude); (b) successful single breath‐hold waveform (maintenance at end‐expiratory baseline); and (c) consistent sequential breath‐hold waveform (high concordance across three attempts) (Figure 4).

**FIGURE 4:**
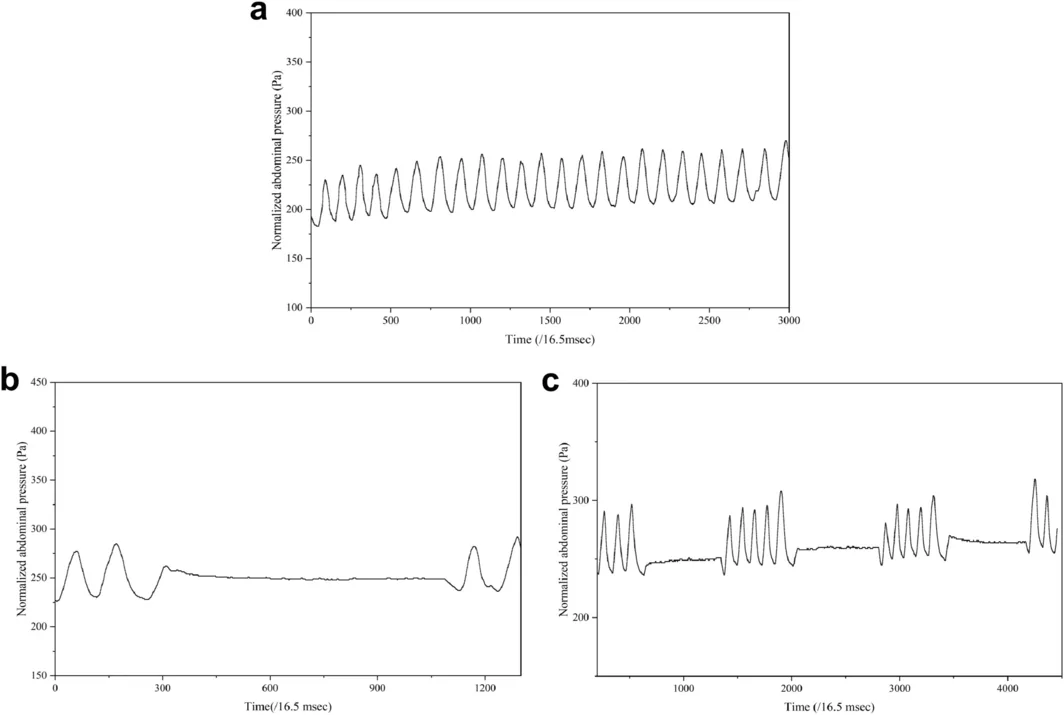
Representative waveforms demonstrating qualified respiratory performance. (a) Stable regular breathing. (b) Adequate single breath‐hold. (c) Consistent serial breath‐holds.

### 2.4 Dynamic Contrast‐Enhanced MRI Protocol

After completing the training in the preparation room, the abdominal belt was taken off. All MRI examinations were performed on a 3 T scanner (uMR 790, United Imaging Healthcare) using a standardized protocol with a 24‐channel phased‐array abdominal coil, with patients positioned supine and feet‐first. The standardized protocol was designed to facilitate a consistent evaluation of respiratory training efficacy. After localizer and non‐contrast sequences, Gd‐EOB‐DTPA was administered intravenously at 0.1 mL/kg (maximum 10 mL) at 1.0 mL/s, followed by a 30‐mL saline flush using a power injector. The start of contrast‐enhanced imaging was synchronized with bolus arrival and the respiratory cycle. Using real‐time bolus tracking with the region of interest placed in the right ventricle, the scan was manually triggered upon detection of a sharp increase in signal intensity and following completion of the breath‐hold instruction. In accordance with routine clinical practice, sequence repetition was permitted when initial breath‐hold performance was suboptimal (e.g., coughing, inadequate breath‐hold duration, or visible motion artifacts). If a breath‐hold sequence failed, it was repeated once immediately following the same breathing instruction. Importantly, all analyses of image quality were based on the initially acquired sequences to reflect the direct impact of training on first‐attempt success. All dynamic phases, including non‐contrast, arterial, portal venous, and transitional, were acquired using an identical breath‐hold 3D T1‐weighted gradient‐echo sequence (TR (Repetition Time)/TE (Echo time)/FA (Turning Angle) = 3.12 ms/1.41 ms/10°). The arterial phase was performed using a serial breath‐hold technique, directly applying the coached breathing pattern. The hepatobiliary phase was also acquired during a breath‐hold. In contrast, T2‐weighted and diffusion‐weighted sequences were obtained during free breathing, serving as internal controls less dependent on respiratory compliance [18, 19]. Detailed technical parameters for all sequences are provided in Table 1.

**TABLE 1:** MRI sequence parameters.

Parameters	Repetition time (TR)/ms	Echo time (TE)/ms	Scan time (ST)/s	Matrix	Field of view (FOV)/mm	slices	Turning angle (FA)	Respiratory control	Scanning sequence and phase				
Cor‐ssfse T2	537	96	13.2	272 × 231	320 × 340	24	120°	Single breath‐hold	①				
Tra‐quick 3D T1	3.12	1.41	14.3	256 × 154	400 × 300	30	10°	Single/Serial breath‐hold(s)	② Plain scan phase	④ Arterial phase	⑤ Portal venous phase	⑥ Transitional phase	⑩ Hepatobiliary phase
Tra‐quick 3D‐wifi T1	3.98	1.38	13.2	320 × 320	400 × 300	34	12°	Single breath‐hold	③				
Cor‐quick 3d‐wifi T1	4.32	1.59	15.9	304 × 242	320 × 340	40	15°	Single breath‐hold	⑦				
Tra‐arms‐T2	3968	91.8	214.8	320 × 320	380 × 380	30	100°	Regular breathing	⑧				
Tra‐EPI‐DWI	3776	52	303	144 × 144	380 × 380	30	90°	Regular breathing	⑨				

*Note:* Serial breath‐holds were used in the dynamic arterial, portal venous, and transitional phase scanning.

### 2.5 Outcome Measures

#### 2.5.1 Patient Cooperation Rate

Patient cooperation during the MRI examination was assessed in real time by the on‐duty MRI technologist, who was blinded to group allocation. Patient cooperation during the breath‐hold sequences was rated using a 3‐point ordinal scale: 3 = full cooperation (perfect adherence to all breathing instructions); 2 = partial cooperation (one instance of irregular breathing or breath‐hold failure); 1 = poor cooperation (two or more instances of irregular breathing or breath‐hold failure). The primary outcome, “patient cooperation rate,” was defined as the proportion of patients achieving “full cooperation” (score = 3) in each group.

#### 2.5.2 Examination Duration

The total examination duration was recorded and segmented into two intervals: (a) Preparation Time, defined as the time from the initiation of standardized respiratory training until the training qualification criteria were met, was manually recorded by the research nurse using a digital stopwatch. This interval encompassed all training‐related activities, including the initial training session and any subsequent reinforcement or correction required for patients who did not achieve proficiency on their first attempt; and (b) Scanning Time, defined as the interval from the initiation of the first sequence to the completion of the last sequence, including any necessary sequence repetitions. When a sequence was repeated due to suboptimal breath‐hold performance, the time required for the repeat acquisition was included in the scanning time to reflect the actual in‐scanner time experienced by the patient. The scanning time was automatically obtained from the MRI scanner's internal log system.

#### 2.5.3 Image Quality

Image quality was evaluated both subjectively and objectively. For the subjective assessment, three board‐certified abdominal radiologists (W.C. with 18 years of experience, X.H. with 20 years of experience, and B.H. with 15 years of experience), all blinded to group allocation and clinical information, independently scored motion artifacts across all contrast‐enhanced phases (arterial, portal venous, transitional, and hepatobiliary) using a validated 5‐point Likert scale [8]: 1, no motion artifacts; 2, minimal artifacts without diagnostic impact; 3, moderate artifacts with minor diagnostic limitations; 4, severe artifacts but images remain interoperable; 5, extensive artifacts rendering images non‐diagnostic. The initial acquisition of each contrast‐enhanced phase was evaluated. In cases where a sequence was repeated due to suboptimal breath‐hold performance, only the first acquisition was used for scoring to reflect the direct impact of the training method on first‐attempt breath‐hold success. All images were anonymized and randomized prior to scoring. The final score for each phase was the average of the three readers' scores. Transient severe motion (TSM) was defined as a mean arterial phase score of ≥ 4 (5‐point scale), with scores ≤ 2 in the non‐contrast and/or other contrast‐enhanced phases (portal venous, transitional, and hepatobiliary).

For the objective assessment, the signal‐to‐noise ratio (SNR) and contrast‐to‐noise ratio (CNR) were calculated for each phase. All ROI measurements were performed by a single trained researcher (G.C. with 12 years of experience in abdominal MRI quantitative analysis), who was blinded to group allocation and all clinical information, to ensure consistency and eliminate inter‐operator variability. As illustrated in Figure 5, three circular regions of interest (ROIs), each with an area of approximately 1.0 cm^2^ (100 mm^2^), were placed in normal liver parenchyma (left lobe, right lobe, and posterior segment), carefully avoiding vessels, bile ducts, and focal lesions. This size was chosen to ensure consistent sampling of homogeneous tissue while avoiding partial volume effects at tissue boundaries. Three corresponding ROIs were placed in the bilateral erector spinal muscle at the same anatomical level. To measure true image noise, a large circular ROI (approximately 20–30 cm^2^) was placed in artifact‐free background air outside the patient, avoiding ghosting artifacts and phase‐encoding direction aliasing. The standard deviation of the signal intensity in this background ROI (SD_air) was recorded as the image noise. From these measurements, the mean signal intensities (SI_liver, SI_muscle) and the background noise (SD_air) were derived. SNR and CNR were then computed using the following standard formulas:

(1) SNR=SI_liver/SD_air

(2) $$\mathrm{CNR}=\left(\mathrm{SI}\_\text{liver}-\mathrm{SI}\_\text{muscle}\right)/\mathrm{SD}\_\mathrm{air}$$

where SI_liver and SI_muscle represent the mean signal intensities of liver parenchyma and erector spinae muscle, respectively, and SD_air represents the standard deviation of background air signal intensity (image noise).

**FIGURE 5:**
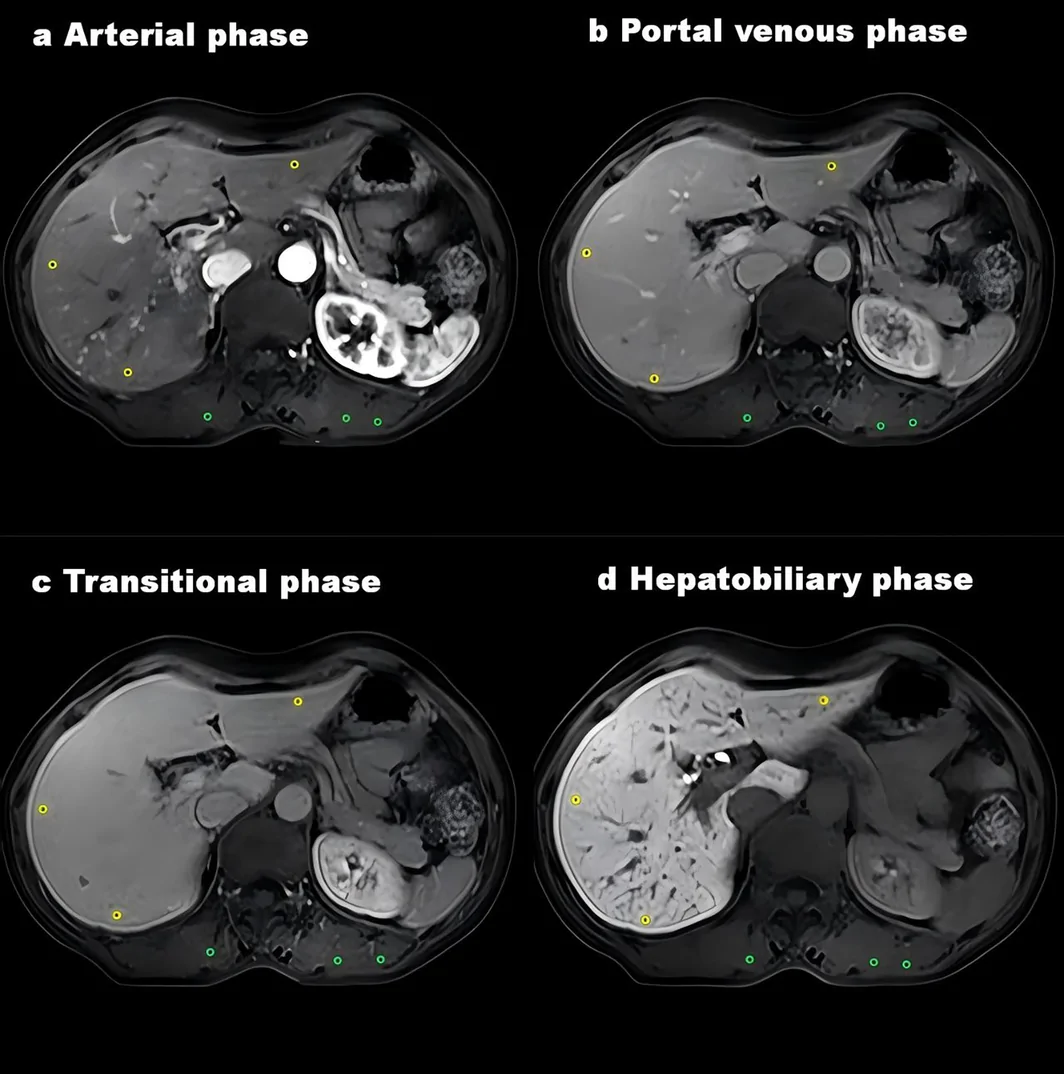
Placement of regions of interest (ROIs) for quantitative measurement of signal‐to‐noise ratio (SNR) and contrast‐to‐noise ratio (CNR). Yellow circles indicate ROIs placed in the liver parenchyma; green circles indicate ROIs in the paraspinal muscle as reference. All ROIs were circular with a fixed area of approximately 1.0 cm^2^ (100 mm^2^). The same ROI size was used for both liver parenchyma and erector spinae muscle measurements.

### 2.6 Statistical Analysis

Sample size estimation was performed using the “Compare 2 Proportions” module of the Power and Sample Size (PASS) software. The calculation was based on the primary outcome of patient cooperation. Based on a review of the literature and our institutional experience with conventional respiratory training, we assumed a “full cooperation” rate of approximately 25% in the control group. We hypothesized that visualized training with real‐time biofeedback would increase this rate to 50%, representing a clinically meaningful improvement. With a statistical power of 80% and a two‐sided alpha level of 0.05, a minimum of 59 participants per group was required. Accounting for an estimated 15% dropout rate, the total planned sample size was 140.

Statistical analyses were performed using SPSS software (version 25.0; IBM Corp.). Continuous variables were tested for normality using the Kolmogorov–Smirnov test. Normally distributed data are presented as mean ± standard deviation (SD) and were compared between groups using the independent Student's *t*‐test. Non‐normally distributed or ordinal data are expressed as median (inter quartile range) and were compared using the Mann–Whitney *U* test. Categorical variables are summarized as frequencies and percentages and were analyzed using the *χ* ^2^ test or Fisher's exact test, as appropriate. Specifically, the patient cooperation score (an ordinal variable) was compared between groups using the Mann–Whitney *U* test, and its association with group allocation was further quantified using the Gamma coefficient. Inter‐observer agreement among the three readers was assessed using the intraclass correlation coefficient (ICC) with a two‐way random effects model for absolute agreement. ICC values were interpreted as follows: < 0.50 indicates poor agreement, 0.50–0.75 indicates moderate agreement, 0.75–0.90 indicates good agreement, and > 0.90 indicates excellent agreement. A two‐tailed *p* value < 0.05 was considered statistically significant for all tests [20].

## 3 Results

### 3.1 Baseline Characteristics

Between June 2023 and October 2024, a total of 243 patients were scheduled for Gd‐EOB‐DTPA‐enhanced liver MRI, of whom 194 met the inclusion criteria. Participants were randomly allocated in a 1:1 ratio to either the control group (*n* = 103) or the experimental group (*n* = 91). As detailed in Figure 1, 4 participants in the control group and 6 participants in the experimental group met the dropout criteria and were excluded during the study. Ultimately, 184 participants (135 male, mean age 53 ± 13 years [range 23–82]) successfully completed the MRI examination and were included in the final analysis. Control (*n* = 99) and experimental (*n* = 85) groups did not show significant differences in previously reported risk factors in the literature, including age (≥ 65 years: 23 [23.23%] vs. 15 [17.65%], *p* = 0.351) and BMI (≥ 25 kg/m^2^: 19 [19.19%] vs. 15 [17.65%], *p* = 0.788), or in other baseline characteristics such as sex, education level, and underlying disease type (Table 2).

**TABLE 2:** Baseline characteristics of patients.

Characteristics	Control group (*n* = 99)	Experimental group (*n* = 85)	*χ* ^2^/*t*	*p*
Age (years)	53.69 ± 14.14	53.26 ± 12.38	0.217	0.829
Age ≥ 65 (years)	23 (23.23%)	15 (17.65%)	0.871	0.351
Gender			0.625	0.429
Male	75 (75.76%)	60 (70.59%)		
Female	24 (24.24%)	25 (29.41%)		
BMI (kg/m^2^)	22.35 ± 3.84	22.33 ± 2.74	0.056	0.956
BMI ≥ 25 (kg/m^2^)	19 (19.19%)	15 (17.65%)	0.072	0.788
Education level			0.258	0.879
Junior high or below	56 (56.57%)	45 (52.94%)		
High school	22 (22.22%)	21 (24.71%)		
College or above	21 (21.21%)	19 (22.35%)		
Disease type			3.805	0.149
Space‐occupying lesion (undetermined)	31 (31.31%)	35 (41.18%)		
Hepatocellular carcinoma	28 (28.28%)	27 (31.76%)		
Others	40 (40.40%)	23 (27.06%)		

Abbreviation: BMI, body mass index.

### 3.2 Patient Cooperation

Patient cooperation during scanning was significantly better in the experimental group than in the control group, with 56.5% (48/85) of patients in the experimental group rated as “full cooperation,” compared with 27.3% (27/99) in the control group. Gamma correlation analysis confirmed a significant, moderate, positive association between group assignment (intervention vs. control) and cooperation score (*γ* = 0.406). The odds ratio for achieving “full cooperation” in the experimental group relative to the control group was 3.46 (95% CI: 1.87, 6.40).

### 3.3 Image Quality

Inter‐observer agreement among the three reviewers was substantial, with average‐measure ICC values of 0.828 (95% CI: 0.780, 0.867) for the arterial phase, 0.750 (95% CI: 0.680, 0.806) for the portal venous phase, 0.764 (95% CI: 0.698, 0.818) for the transitional phase, and 0.770 (95% CI: 0.706, 0.822) for the hepatobiliary phase, indicating good to excellent agreement.

The median values of averaged subjective motion artifact scores were significantly lower in the experimental group than in the control group for the arterial (2 [IQR: 1.7, 2] vs. 2 [2, 2]), portal venous (2 [1.7, 2] vs. 2 [2, 2]), and transitional phases (2 [1.7, 2] vs. 2 [2, 2]). No significant difference was observed in the hepatobiliary phase (2.00 [IQR: 1.00, 2.00] vs. 2.00 [1.70, 2.00]; *p* = 0.060). To assess the proportion of patients with diagnostically acceptable images, motion artifact scores were dichotomized into “acceptable” (scores ≤ 3) versus “unacceptable” (scores > 3). The proportion of patients with acceptable images was consistently higher in the experimental group across all phases (Arterial: 97.6% vs. 94.9%, *p* = 0.454; Portal venous: 100% vs. 98.0%, *p* = 0.500; Transitional: 98.8% vs. 99.0%, *p* > 0.999; Hepatobiliary: 100% vs. 98.0%, *p* = 0.500). However, none of these differences reached statistical significance. Table 3 summarizes the score frequencies from three reviewers for four imaging phases in both groups.

**TABLE 3:** Motion artifact score frequencies from three reviewers for four imaging phases.

Phase	Motion artifact score	Control (*n* = 99)			Experimental (*n* = 85)		
		Reviewer 1	Reviewer 2	Reviewer 3	Reviewer 1	Reviewer 2	Reviewer 3
Arterial	1	7 (7.07%)	3 (3.03%)	9 (9.09%)	22 (25.88%)	17 (20.00%)	19 (22.35%)
	2	77 (77.78%)	80 (80.81%)	83 (83.84%)	58 (68.24%)	62 (72.94%)	64 (75.29%)
	3	9 (9.09%)	10 (10.10%)	2 (2.02%)	2 (2.35%)	4 (4.71%)	1 (1.18%)
	4	4 (4.04%)	5 (5.05%)	5 (5.05%)	3 (3.53%)	2 (2.35%)	1 (1.18%)
	5	2 (2.02%)	1 (1.01%)	0 (0.00%)	0 (0.00%)	0 (0.00%)	0 (0.00%)
Portal venous	1	11 (11.11%)	2 (2.02%)	7 (7.07%)	21 (24.71%)	20 (23.53%)	16 (18.82%)
	2	81 (81.82%)	88 (88.89%)	88 (88.89%)	63 (74.12%)	64 (75.29%)	68 (80.00%)
	3	5 (5.05%)	7 (7.07%)	4 (4.04%)	1 (1.18%)	1 (1.18%)	1 (1.18%)
	4	2 (2.02%)	2 (2.02%)	0 (0.00%)	0 (0.00%)	0 (0.00%)	0 (0.00%)
	5	0 (0.00%)	0 (0.00%)	0 (0.00%)	0 (0.00%)	0 (0.00%)	0 (0.00%)
Transitional	1	6 (6.06%)	5 (5.05%)	7 (7.07%)	14 (16.47%)	15 (17.65%)	19 (22.35%)
	2	86 (86.87%)	86 (86.87%)	90 (90.91%)	70 (82.35%)	67 (78.82%)	64 (75.29%)
	3	6 (6.06%)	7 (7.07%)	2 (2.02%)	0 (0.00%)	3 (3.53%)	2 (2.35%)
	4	1 (1.01%)	1 (1.01%)	0 (0.00%)	1 (1.18%)	0 (0.00%)	0 (0.00%)
	5	0 (0.00%)	0 (0.00%)	0 (0.00%)	0 (0.00%)	0 (0.00%)	0 (0.00%)
Hepatobiliary	1	21 (21.21%)	25 (25.25%)	15 (15.15%)	26 (30.59%)	29 (34.12%)	15 (17.65%)
	2	73 (73.74%)	63 (63.64%)	83 (83.84%)	58 (68.24%)	53 (62.35%)	68 (80.00%)
	3	3 (3.03%)	9 (9.09%)	1 (1.01%)	1 (1.18%)	3 (3.53%)	2 (2.35%)
	4	2 (2.02%)	1 (1.01%)	0 (0.00%)	0 (0.00%)	0 (0.00%)	0 (0.00%)
	5	0 (0.00%)	1 (1.01%)	0 (0.00%)	0 (0.00%)	0 (0.00%)	0 (0.00%)

For the quantitative assessment of image quality, the experimental group demonstrated significantly higher SNR and CNR in both the arterial phase (SNR: 225.56 [IQR: 180.39, 264.96] vs. 183.50 [154.00, 220.56]; CNR: 67.53 [49.04, 89.49] vs. 57.39 [44.44, 73.11]) and the transitional phase (SNR: 340.44 [281.00, 387.28] vs. 300.73 [250.29, 358.14]; CNR: 172.81 [141.59, 198.78] vs. 144.80 [122.05, 190.40]). No significant between‐group differences in SNR or CNR were observed for the portal venous (SNR: *p* = 0.367; CNR: *p* = 0.228) or hepatobiliary phases (SNR: *p* = 0.335; CNR: *p* = 0.176).

The incidence of transient severe motion (TSM) was lower in the experimental group (1.18%, 1/85) compared to the control group (5.05%, 5/99), although this difference was not statistically significant (*p* = 0.219). The odds ratio for TSM in the control group relative to the experimental group was 4.468 (95% CI: 0.512–39.020). The risk difference was −3.87% (95% CI: −9.96%, 2.22%).

### 3.4 Examination Duration

The visualized respiratory training significantly improved pre‐scan workflow efficiency. As shown in Table 4, preparation time was significantly shorter in the experimental group than in the control group (14.40 ± 2.36 vs. 16.16 ± 2.20 min; mean difference: −1.76 min; 95% CI: −2.42, −1.10), contributing to a significantly reduced total examination duration (39.04 ± 3.52 vs. 41.12 ± 3.81 min; mean difference: −2.08 min; 95% CI: −3.16, −1.00). In the experimental group, total scanning time—including all sequence repetitions—did not differ significantly between groups (24.60 ± 1.60 vs. 24.89 ± 2.21 min; mean difference: −0.29 min; 95% CI: −0.87, 0.29; *p* = 0.329).

**TABLE 4:** Comparison of examination duration.

Examination duration (min)	Control (*n* = 99)	Experimental (*n* = 85)	Mean difference (95% CI)	*p*
Preparation time	16.16 ± 2.20	14.40 ± 2.36	−1.76 (−2.42, −1.10)	< 0.001
Scanning time	24.89 ± 2.21	24.60 ± 1.60	−0.29 (−0.87, 0.29)	0.329
Total examination time	41.12 ± 3.81	39.04 ± 3.52	−2.08 (−3.16, −1.00)	< 0.001

## 4 Discussion

The implementation of a visualized respiratory training system led to a significant increase in patient cooperation during Gd‐EOB‐DTPA‐enhanced liver MRI, which constitutes the most direct evidence of its effectiveness. Patients in the experimental group demonstrated markedly better adherence to breathing instructions than those in the control group, with over twice the proportion rated as “fully cooperation.” This improvement likely stems from two synergistic mechanisms facilitated by the visual biofeedback system. First, the real‐time translation of abstract verbal commands into an intuitive visual waveform aligns with Dual Coding Theory, wherein simultaneous visual and verbal processing enhances comprehension and retention [21]. This enabled patients to actively self‐correct their breathing patterns, a principle supported by biofeedback studies in other fields such as meditation training [22]. Second, the protocol included simulated scanner acoustics (equipment noise, voice prompts, and breath‐hold abjurations), providing pre‐scan familiarization that likely mitigated situational anxiety [12]. Reduced anxiety and improved predictability of the scanning process have been linked to better patient cooperation and, ultimately, higher image quality [13].

The observed gain in patient cooperation during the breath‐hold sequences likely contributed to better control of respiratory motion artifacts. Subjective ratings showed significantly lower artifact scores in the experimental group across the arterial, portal venous, and transitional phases. Objective metrics also showed significant improvements in both SNR and CNR for the arterial (the narrowest temporal acquisition window and the phase most vulnerable to respiratory motion) and transitional phases. It is well recognized that respiratory motion can degrade image quality by introducing phase errors across k‐space, leading to signal misregistration, blurring, and structured noise, all of which can reduce SNR [23]. Furthermore, motion degrades CNR not only by reducing the signal from the liver but also by diminishing the signal difference between the liver and reference tissue (e.g., erector spinae muscle), while simultaneously elevating background noise levels [24]. In the experimental group, real‐time visual feedback enabled patients to achieve more consistent breath‐holds, minimizing motion‐induced signal loss and preserving tissue contrast. This effect was most pronounced in the arterial phase—the narrowest and most motion‐sensitive temporal window—where the intervention provided the greatest benefit. The transitional phase, acquired approximately 120 s post‐injection, represents a period of relative hemodynamic stability; the significant improvements observed in this phase may be attributable to the cumulative effect of repeated breath‐hold practice during the scan, leading to sustained benefit throughout the dynamic acquisition [25]. Additionally, successful completion of the demanding arterial phase may reinforce patient confidence and cooperation for subsequent phases. The lack of significant inter‐group differences in the portal venous and hepatobiliary phases may be attributable to several factors. The portal venous phase represents a transitional period when contrast dynamics are still evolving but the acute hemodynamic changes have subsided. For the hepatobiliary phase, which relies on hepatocyte‐specific uptake rather than breath‐hold performance [18, 19], the high intrinsic signal intensity may mask residual motion effects, making it less sensitive to the benefits of improved breath‐hold consistency.

When motion artifact scores were dichotomized into diagnostically acceptable (scores ≤ 3), versus unacceptable (scores > 3), no significant between‐group differences emerged. This finding reflects the overall high image quality achieved in both groups under structured training, with over 94% of patients already in the acceptable range. The full score distribution reveals that the intervention's primary benefit was a shift toward better scores within the acceptable range—more patients achieved the best score (score = 1) and fewer received intermediate scores (score = 3)—rather than a reduction in the already low rate of unacceptable images. Dichotomization discards this clinically meaningful distinction, reducing statistical power [26]. The consistent numerical advantage in acceptable image rates across all phases, combined with the significant improvements detected by ordinal analysis, supports the conclusion that visualized training enhances image quality in a clinically meaningful way.

The incidence of transient severe motion (TSM) in both groups was lower than the average rate reported in the literature (~13%) [6], underscoring the inherent value of any structured respiratory training. This observation reflects our study design: both groups received standardized pre‐scan training, which may represent a more intensive level of preparation. Although the visualized training showed a notable numerical reduction compared to conventional training, this difference did not reach statistical significance, and the wide confidence interval for the risk difference underscores the limited precision of this estimate. This reflects the low event rate and limited sample size; the study was powered for the primary outcome of patient cooperation rather than TSM, and thus lacked sufficient power to detect a statistically significant difference for this secondary outcome. Future investigations with larger cohorts are warranted to definitively assess the impact of visualized training on TSM reduction.

Beyond image quality, the visualized system also improved overall workflow efficiency. Although the experimental group showed better patient cooperation, scanning time did not differ significantly between groups; total examination duration was comparable between groups; the main efficiency gain occurred during preparation rather than scanning. Despite the need for initial device setup by research nurses, the experimental group had a significantly shorter preparation time. This counterintuitive result stemmed from the visual feedback system, which allowed rapid patient proficiency, fewer training cycles, and targeted real‐time correction of breathing irregularities without full retraining. In contrast, conventional subjective training with abdominal palpation in the control group required more frequent and complete retraining cycles. The higher efficiency of objective waveform‐guided assessment fully offset the device setup time [27], and visual feedback accelerates learning. In terms of local clinical practice, equivalent scanning time in both groups likely stems from overwhelming workloads. To complete required examinations on schedule, technologists prioritize scan completion once images are diagnostically adequate and avoid repeated acquisitions for better image quality. This aligns with motion artifact scores: no intergroup difference was observed in the proportion of diagnostically acceptable images, whereas the experimental group had more high‐quality images assigned “no motion artifacts (score = 1).” Collectively, these findings demonstrate that the intervention improves workflow efficiency primarily by optimizing pre‐scan preparation, with time saved from reduced retraining outweighing the initial setup burden.

The visualized training system offers notable clinical benefits, but its real‐world adoption also depends on practical feasibility. In this study, the training was administered by the same radiology nurses who provided conventional instructions, and the experimental group actually had shorter preparation times, indicating that the system does not increase staffing demands. The hardware consists of a reusable pressure‐sensor belt and a mobile terminal (e.g., a mobile device), which can be deployed on existing devices, keeping incremental costs low. Moreover, training was conducted in standard preparation rooms without requiring dedicated space. These characteristics suggest that the system can be integrated into routine workflows with minimal additional resources. Future studies evaluating cost‐effectiveness and scalability across different institutional settings would further inform implementation decisions.

### 4.1 Limitations

First, its single‐center design, single MRI vendor, single field strength, and limited sample size may have reduced the generalizability of the results. Second, the study included only patients undergoing first‐time Gd‐EOB‐DTPA‐enhanced liver MRI; findings may not directly apply to those with prior scan experience, who may have different baseline cooperation levels. Third, randomization was performed using a pragmatic method based on the parity of the examination number rather than computer‐generated allocation. Although baseline characteristics were balanced between groups, this approach could potentially introduce selection bias. Fourth, the primary outcome (patient cooperation) was assessed by the on‐duty MRI technologist using a 3‐point ordinal scale. Although the technologist was blinded to group assignment, the assessment was subjective. This may have introduced some measurement bias. Finally, while we implemented blinding for patients, technologists, and image evaluators, the personnel administering the respiratory training could not be blinded due to the nature of the intervention, which may have influenced training delivery.

## 5 Conclusion

Visualized respiratory training improved patient cooperation compared with conventional training. This improvement was associated with better motion artifact scores in the arterial, portal venous, and transitional phases, and with higher SNR and CNR in the arterial and transitional phases. The training also reduced preparation time and total examination duration. However, the direct link between improved cooperation and objective image quality metrics is not fully established. In addition, the reduction in TSM and scanning time was not statistically significant. Therefore, while visualized training shows promise for enhancing patient cooperation and workflow efficiency, its clinical benefits remain to be confirmed in further studies.

## Funding

This study was supported by Research Fund of Tongji Hospital (No. 2023D23).
